# Supplementary material for: Phase Separation of SARS-CoV-2 Nucleocapsid Protein with TDP-43 Is Dependent on C-Terminus Domains
Source: Int J Mol Sci. 2024 Aug 12;25(16):8779. doi: 10.3390/ijms25168779 (PMC11354357; doi:10.3390/ijms25168779)
Supplement: Supplementary file 1 [file ijms-25-08779-s001.zip › Supplementary Table S1.pdf]

Supplemental Table S1. TDP-43 intramolecular interaction properties within the TDP-43-N protein heteropolymers in the presence and absence of RNA.

Heteropolymer Complex	Interface	Structure 1 (Chain)	Structure 2 (Chain)	Δ^iG (kcal/mol)	Total Interface Area (Å²)	Interface TDP-43 IDR Buried Surface Area (Å²)*	Total Complex TDP-43 IDR Buried Surface Area (Å²)*
No RNA	1	TDP-43 A	N Protein A	-14.0	3186.2	1529.9	4129.8
	2		N Protein B	-6.1	434.4	422.4	
	3		N Protein C	-1.8	598.4	0	
	4		N Protein D	-2.9	2058.5	111.9	
	5		TDP-43 B	-0.5	85.5	0	
	6	TDP-43 B	N Protein A	-1.9	300.1	0	
	7		N Protein B	-1.5	2220.5	84.6	
	8		N Protein C	-15.4	2277.0	1674.7	
	9		N Protein D	0.2	1350.6	306.3	
	5		TDP-43 A	-0.5	85.5	0	
70-mer RNA	10	TDP-43 A	N Protein A	-2.6	1536.9	781.1	18101.9
	11		N Protein B	-27.4	2238.5	1975.0	
	12		N Protein C	-20.3	1538.3	1531.5	
	13		N Protein D	-17.1	2507.9	2554.1	
	14		TDP-43 B	-19.0	3275.5	1730.1	
	15		RNA A	-9.4	505.3	91.8	
	16		RNA B	-14.4	719.5	351.7	
	17	TDP-43 B	N Protein A	-20.7	1583.6	1569.5	
	18		N Protein B	-16.6	2495.8	2523.6	
	19		N Protein C	-2.5	1587.5	796.7	
	20		N Protein D	-27.7	2234.8	2007.7	
	14		TDP-43 A	-19.0	3275.5	1723.8	
	21		RNA A	-13.2	690.5	335.0	
	22		RNA B	-9.1	538.3	130.3	

*- IDR buried surface area is in reference to Structure 1 $TDP\text{-}43_{263\text{-}414}$.
