# Supplementary figures and images for: Phase Separation of SARS-CoV-2 Nucleocapsid Protein with TDP-43 Is Dependent on C-Terminus Domains
Source: Int J Mol Sci. 2024 Aug 12;25(16):8779. doi: 10.3390/ijms25168779 (PMC11354357; doi:10.3390/ijms25168779)

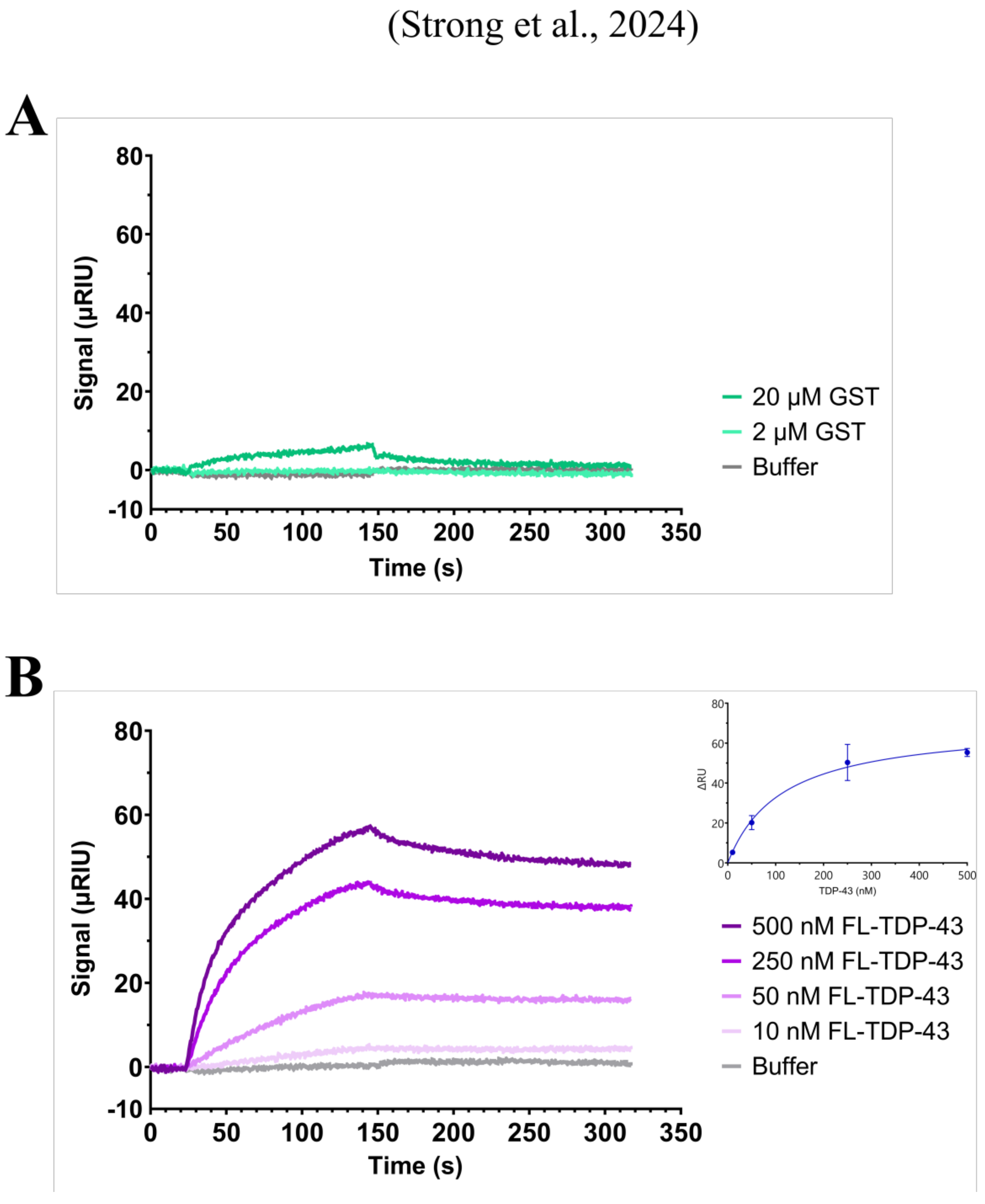

Supplement: Supplementary file 1 [file ijms-25-08779-s001.zip › Strong et al Supplementary Figure 2.tiff]

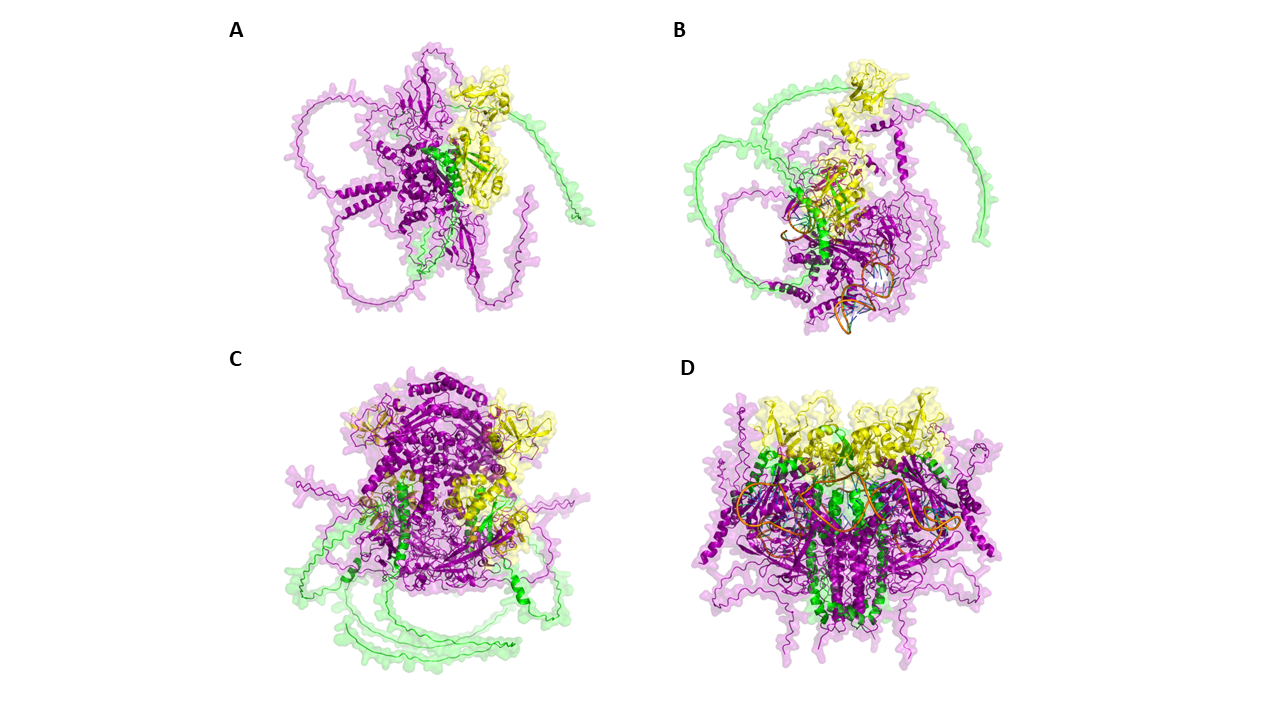

Supplement: Supplementary file 1 [file ijms-25-08779-s001.zip › Supplemental figure S3.TIF]
